# Preliminary Study of Heavy Metals in Low-Cost Jewelry Items Available in Nigerian Markets

**DOI:** 10.5696/2156-9614-10.28.201202

**Published:** 2020-11-19

**Authors:** Gilbert U. Adie, Esther O. Oyebade, Boluwatife M. Atanda

**Affiliations:** Department of Chemistry, Faculty of Science, University of Ibadan, Ibadan, Nigeria

**Keywords:** low-cost jewelry, Heavy metals, European Union safety limit, Ibadan, Nigeria

## Abstract

**Background.:**

Many developing countries either lack or have weakly enforced regulations on imported goods. A high percentage of low-cost jewelry items in Nigeria are imported from abroad. There is concern about the levels of heavy metals present in these products.

**Objectives.:**

The present study examined the levels of lead (Pb), cadmium (Cd), chromium (Cr) and nickel (Ni) in inexpensive jewelry purchased from retail wholesale shops in Ibadan metropolis, southwestern Nigeria.

**Methods.:**

One hundred (100) assorted jewelry items were digested in dilute nitric acid solution followed by atomic absorption spectrophotometric analysis.

**Results.:**

Out of the total number of jewelry items analyzed, 12% of them had Pb concentrations above European Union (EU) safety limits. Also, 63%, 42% and 62% of items had Cd, Cr and Ni average concentrations above their EU limits. Notably, 3%, 27% and 8% of the items had Pb, Cd and Cr concentrations over 10^3^ times above their EU limits Overall, Cd showed the highest average level in rings (256,952 mg/kg) followed by bracelets (60,627 mg/kg) and earrings (54,388 mg/kg). All metals in solid bangles were within their EU guidelines.

**Conclusions.:**

Given the significant deleterious impacts of these metals on human health, low-cost jewelry poses a serious potential threat to users' health. Policies to guarantee the safety of low-cost jewelry items must be established and enforced.

**Competing Interests.:**

The authors declare no competing financial interests.

## Introduction

Human exposure to heavy metals is on the rise and requires both global and regional attention.^1^ In addition to background and natural sources, like rock weathering, volcanoes, etc., there are many other anthropogenic sources of heavy metal pollution, including crude recycling of used lead acid batteries and end-of-life electronic wastes, wear and tear of vehicular bodies and tires, untreated industrial effluents and releases from metallurgical industries. Another overlooked source, especially in developing countries, is metal released from low-cost jewelry items through either dermal contact or ingestion. Children and women are more vulnerable to exposure to toxic metals because of their behavior and physiological makeup.^2–3^ Women are more prone to wear jewelry items than men, and therefore are at higher risk of exposure, especially through the skin. Children are even more vulnerable than adults as many of them normally play with their jewelry items by mouthing them, and therefore are at risk of the metals being extracted by the saliva or swallowing, in addition to dermal exposure. There has also been evidence of the transfer of toxic metals from mothers' breast milk to their children.^4^

Many heavy metals have irreversible deleterious effects on young children. For instance, lead (Pb) is known to reduce intelligence quotient, especially in children, induce kidney damage and can be fatal with high dose exposures.^3,5^ Other acute symptoms from Pb poisoning can include loss of appetite, headache, hypertension, abdominal pain, arthritis, etc.^6^ A possible lead toxicity mechanism could occur when Pb^2+^ replaces other bivalent cations like calcium (II), magnesium (II) and iron (II) and monovalent cations like sodium (I), which eventually disrupts the biological metabolism of the cell. This causes significant changes in various biological processes. Substitution of calcium in ultra-trace concentrations affects protein kinase carbon which regulates neural excitation and memory storage.^1^ Cadmium (Cd) has been found to easily replace zinc, thereby inhibiting its activities as a free radical as well as binding to cysteine–rich protein in cells and can lead to deficiency in iron (II).^7^ Cadmium exposure has been observed to cause osteoporosis (skeletal damage) and kidney damage in both humans and other animals.^8^ The metal is known for causing the common health hazard called ‘*Itai Itai* disease' first noticed in Japan around 1912.^9^ Chromium (Cr) exists in different valent states with Cr(III) the most stable, less toxic and beneficial in glucose metabolism in humans, while Cr(VI) is highly unstable, toxic and carcinogenic in nature. Nasal ulcer is common among workers that deal with Cr-containing compounds.^1^ Nickel (Ni) is not an essential metal and quite toxic, especially in aquatic environments. Nickel does not break down easily in the environment and can bio-accumulate in living systems for many years even at low level exposures. The metal is usually regarded as a xenobiotic substance in human systems with risks of carcinogenicity.^10^

There have been numerous cases of heavy metal poisoning through the ingestion of jewelry; in 2006 in Minnesota, United States of America (USA), there was a case of fatal Pb poisoning after a child ingested a Pb-contaminated charm; there was another instance of Pb poisoning in Oregon after a young child consumed a necklace medallion.^11^ These incidents led to the recall of several million Pb- and Cd-containing jewelry items on the market.^3,12,13^ Other studies on human exposure to toxic metals in jewelry items have been reported.^14–17^

Weidenhamer and Clement reported that one of the main sources of Pb in jewelry items could be from the use of recycled electronic wastes and battery Pb as a source material.^18–20^ They found Pb, tin, and copper composition in some jewelry items similar to that in the solder of circuit boards, as well as Pb/antimony composition similar to that in Pb batteries. Elemental Pb is usually added to paints as a coloring agent in the production of toys and low-cost jewelry items to prevent free radicals from reacting to form acidic media.^5^ Furthermore, heavy metals are sometimes intentionally added to jewelry items to serve as coating agents, to lower the cost of manufacturing (as many of them are cheap), for easier workability, to provide shiny surfaces and to make jewelry items heavier in attempt to mimic superior quality products.

**Abbreviations**
*AAS*: Atomic absorption spectrometer
*BDL*: Below detection limit
*EU*: European Union

Nigeria, like most developing countries, imports all kinds of jewelry items from abroad, mainly from China, the USA, Brazil, Italy, India, and the United Kingdom, etc. There is high demand for consumer goods in Nigeria as a result of increasing population and lack of industries to produce products to meet this demand. Many of these goods arriving in Nigeria do not meet the accepted guidelines due to either the lack of or weak enforcement of policies/regulations guiding the qualities of imported goods. The levels of toxic metals present in low-cost jewelry items sold in Nigerian markets is of concern as there is paucity of data within the African region. Therefore, the present study aimed to examine the levels of heavy metals in low-cost jewelry items sold in Ibadan, southwestern Nigeria and to determine the safety of these products.

## Methods

**Figure 1:**
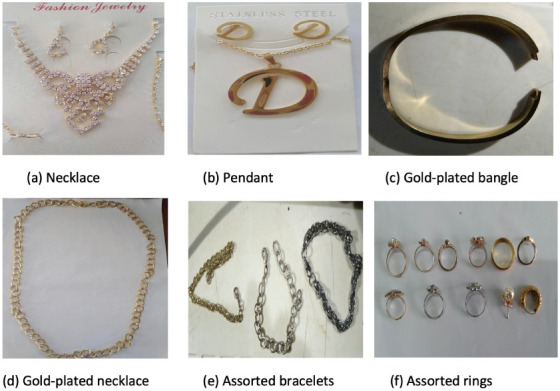
Selected samples of studied jewelry items

The present study was conducted between February and June 2019. A total of 100 low-cost jewelry items which were metallic in nature were purchased from different retail and wholesale stores in Agbowo, Bodija, Mokola, Dugbe and Agbeni markets in Ibadan, southwestern Nigerian. Table 1 presents a summary of the jewelry item types, weight and cost ranges (all jewelry items were under $2 US dollars) while Figure 1 provides photos of some of the items studied. All items were stored separately in small polythene bags and labelled accordingly prior to analyses.

**Table 1:** Jewelry Items

**Sample type**	**Total no.**	**Color**		**Weight(g)**		**Price range (Naira)**	Price range** (USD)

		**Gold**	**Silver**	**Average**	**Range**		
Bangle*	6	6	0	3.723	3.221–4.404	200–300	0.54–0.8
Earring	33	16	17	1.72	0.043–11.52	50–300	0.13–0.8
Bracelet*	4	4	0	8.24	3.123–11.02	150–200	0.40–0.5
Necklace	36	25	11	17.38	0.6113–58.73	100–600	0.27–1.6
Pendant	9	9	0	4.26	2.112–9.950	200–300	0.54–0.8
Ring	12	8	4	2.176	0.496–4.039	100–300	0.27–0.8

^*^Bangle is defined as a solid ring worn around the wrist. Bracelet is a chain worn around the wrist.

^**^Exchange rate: 1 US dollar = 371.235 naira (exchange rate as at 26/03/2020).

Source^21^

### Sample preparation and chemical analyses

Jewelry items weighing ≤5.0 g were digested whole, while those weighing >5.0 g were carefully cut using acid washed stainless handheld pliers into pieces and a piece weighing ≤5.0 g was digested. The plier was cleaned after every cut to prevent cross contamination. The method used by Weidenhamer and Clement was adopted with slight modification.^18^ Each weighed item was placed in a pre-cleaned Pyrex beaker and soaked overnight with 10 mL of 50% (v/v) Analar grade nitric acid. Each sample was then heated on a thermostatic electric hot plate in the hood until complete dissolution occurred. The digests were allowed to cool, then filtered using Whatman filter paper and made up to mark in an appropriate volumetric flask. The concentrations of Pb, Cd, Cr and Ni in the digests were then determined using Perkin-Elmer AAnalyst 200 atomic absorption spectrometer (AAS) equipped with single element hollow cathode lamp and 10 cm air–acetylene burner flame. Blank samples were also carried out using the same method to check reagent impurities.

All reagents used were of Analar grade. All glassware were cleaned by soaking in diluted nitric acid solution overnight and thoroughly rinsed with distilled water prior to use. This was necessary to remove any adsorbed metals on the walls of the glassware. Blanks were used to check reagent contamination with metals of interest. Furthermore, 10% of the samples were duplicated to check precision.

A preliminary analysis including double blind samples was carried out to ensure proper functioning of the AAS and to validate the repeatability of the results in order to determine the results generated from the instrument were accurate.

## Results

A summary of total concentrations of Pb, Cd, Cr and Ni in all jewelry items is presented in Table 2. The European Union (EU) safety limits for each metal are also included in the table for comparison.^22^ Cadmium showed the highest average concentration of 59 125 mg/kg with concentrations in all items ranging from below detection limit (BDL)-922 000 mg/kg. Lead showed the lowest average concentration of 950 mg/kg with concentrations in all items ranging from BDL-24 500 mg/kg. Chromium had an average of 10 456 mg/kg and ranged from BDL-62 008 mg/kg, while Ni had an average of 9511 mg/kg and ranged from BDL-49 751 mg/kg. It is noteworthy to mention that the metal concentrations detected in the blanks were subtracted from the results before computation. Furthermore, blind and duplicate samples to validate the accuracy and precision, respectively of AAS procedure, were within the allowable limits of 10% for the AAS technique.

**Table 2:** Summary of Metal Concentrations (mg/kg) in Jewelry Items

**Metal**	**No. of samples analyzed**	**Average**	**Range**	**EU limit (2013)**	**% within EU limit**	**% 10^1^–10^2^ × EU limit**	**% 10^2^–10^3^ × EU limit**	**% >10^3^ × EU limit**
Pb	100	950	BDL-24500	160	88 (BDL=45)	3	6	3
Cd	100	59125	BDL-922000	17	37 (BDL=32)	17	19	27
Cr	50	10456	BDL-62008	460	58 (BDL=40)	0	34	8
Ni	50	9511	BDL-49751	930	38 (BDL=6)	24	38	0

Below detection limit: Pb = 0.04, Cd = 0.10, Cr = 0.04, Ni = 0.05 (mg/L).

### Summary of metal concentrations in six different types of jewelry items

Table 3 shows the six different types of jewelry item in the present study: bangle (6 items), earring (33 items), bracelet (4 items), necklace (36 items), pendant (9 items) and ring (12 items).

**Table 3:** Summary of Metal Concentrations (mg/kg) Across Jewelry Item Types

**Jewelry item**	**Metal**	**Total no. samples**	**No. analyzed**	**Mean**	**Range**	**EU guideline**	**No. higher than EU guideline**	**% higher than EU guideline**
Bangle	Cr	6	0	-	-	-	0	-
	Cd		6	16.7	BDL-99.8	17	1	16.7
	Pb		6	58.5	BDL-322	160	1	16.7
	Ni		0	-	-	-	0	-
Earring	Cr	33	11	3138	BDL-21877	460	2	18.2
	Cd		33	54388	BDL-804000	17	16	48.5
	Pb		33	473	BDL-922	160	2	6.1
	Ni		11	6166	BDL-11177	930	6	54.5
Hand chain	Cr	4	0	-	-	-	-	-
	Cd		4	60627	96.6–101663	17	4	100
	Pb		2	3081	BDL-3766	160	2	100
	Ni		4	215	8.24–818	930	0	0
Necklace	Cr	36	28	15277	BDL-62008	460	18	64.3
	Cd		36	8640	BDL-115250	17	28	77.8
	Pb		36	252	BDL-3862	160	3	8.3
	Ni		28	12398	BDL-49751	930	21	75
Pendant	Cr	9	3	20143	13372-29202	460	3	100
	Cd		9	71.2	BDL-438	17	4	44.4
	Pb		9	2737	BDL-24500	160	1	11.1
	Ni		3	19031	11740-23957	930	3	100
Ring	Cr	12	4	35.6	BDL-70.8	460	0	0
	Cd		12	296952	BDL-922000	17	10	83.3
	Pb		12	3269	BDL-19000	160	3	25.0
	Ni		4	651	12.8-2508	930	1	25.0

### Comparison of Pb and Cd (mg/kg) in gold- and silver-plated jewelry items

**Figure 2:**
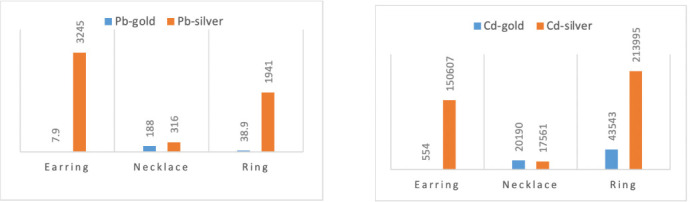
(a) Comparison of average Pb concentrations in gold-plated and silver-plated jewelry items; (b) Comparison of average Cd concentrations in gold-plated and silver-plated jewelry items

The concentrations of Pb and Cd in earring, necklace and ring items that were plated with gold and silver were compared and are shown in Figure 2a and 2b.

## Discussion

As shown in Table 2, the concentrations of the metals in all items followed the sequence Cd> Cr>Ni>Pb. Comparing the results with EU safety guidelines as shown in Table 2, Cd seemed to pose the highest risk with 63% of 100 items having a concentration higher than the EU guideline of 17 mg/kg. Furthermore, over 27% of the items showed a Cd concentration 10^3^ times over the EU guideline. Lead posed the lowest risk with over 88% of 100 items within the EU guideline of 160 mg/kg and 45% of the items below the detection limit of 0.04 mg/L. However, two earrings and one bangle had a Pb level over 10^3^ times the EU guideline of 160 mg/kg. Chromium and Ni levels ranged between the Cd and Pb concentrations. The higher average Cd content in the items in the present study supports the report by Becker *et al.* which indicated that the use of Pb-contaminated materials to produce toys and jewelry items was decreasing as manufacturers were shifting attention from Pb to Cd and other metals as a result of stricter regulations on Pb due to its toxicity, especially in children.^23^ Furthermore, the 2008 Annual Report on the Cadmium Market reported that 75% of the Cd produced in China was used for Ni-Cd batteries which was reused at end-of-life to make low-cost toys and jewelry items.^24^ The concentrations of Cr and Ni were 42% and 62% higher than their EU guideline, respectively, of 50 samples studied *(Table 2).* All the average metal contents in the studied items were higher than those reported in a similar study by Cui *et al.* carried out on 13 metallic toy and jewelry items in China.^25^ Another study reported variable metal concentrations within the ranges of the present study.^5^

The mean bioaccessibility of metals in low-cost children's toys and jewelry items reported by Cui *et al.* for saliva and hydrogen chloride extracts compared with total metal concentrations were 2.57 and 6.45% (Ni), 7.59 and 8.56% (Cd), 1.78 and 22.6% (Pb) and 1.94 and 24.0% (Cr).^25^ Our study did not report bioaccessibility tests of the metals, however judging from the concentrations of metals obtained compared with the % bioaccessibility (concentration of metal likely to be available for intake by biological cells) of metals in a study by Cui *et al.*, it is thought that there could be a possibility of potential risk associated with mouthing or ingesting the jewelry items, especially by children.^25^ This calls for close monitoring of children wearing these jewelry items by adults as well as the need for the government to formulate and enforce policies and regulations to ensure the quality of low-cost jewelry items arriving in Nigeria.

As shown in Table 3, ring items showed the highest average Cd concentration of 296 952 mg/kg, which ranged from BDL-922 000 mg/kg, and had an average concentration higher than 10^4^ -fold the EU guideline. The items also showed an average Pb concentration of 3269 mg/kg (more than ×10 the EU safety guideline). The metal concentrations exhibited in the ring items are worrying considering that most adults/children wear them for long periods of time compared with other jewelry types and mouth contact may be higher. Chromium and Ni were within their EU regulatory guidelines for the ring items. The average Cd level was many times higher than the EU limit in almost all jewelry items, except for bangles. The mean concentrations of all the metals in pendants, earrings and necklaces indicated levels many times higher than the EU safety guidelines *(Table 3).* This may indicate that manufacturers of these products are not adhering to existing global guidelines regarding the amount of these metals allowed in consumer products.

Both Pb and Cd had significantly greater levels in silver-plated earring and ring items compared to gold items. Necklace items had comparable levels. Overall, silver-plated items pose more risk than gold-plated items.

## Conclusions

The concentrations (mg/kg) of Pb, Cd, Cr and Ni in over 100 samples of low-cost jewelry items were examined to assess their safety when worn. All metals showed varying percentage levels higher than their European Union safety limit. Notably, 3%, 27% and 8% of the items analysed had Pb, Cd and Cr concentrations over 10^3^ times above their EU limits. The results suggest that manufacturers are shifting from Pb to other metals like Cd perhaps due to stricter regulations. Cadmium was observed to have the highest concentration in rings, while all the metals in hand bangles were within their EU levels as shown in Table 3.

Although a bioaccessibility study was not carried out to identify the real time leaching of metals into the human system, previous studies suggest that the average concentrations of these metals can have serious deleterious effects on users, especially children, when mouthed or ingested. Policies on the recommended levels of a wide range of toxic heavy metals in low-cost jewelry items arriving in Nigeria need to be put in place and enforced to protect users.
